# Genotype Fingerprints Enable Fast and Private Comparison of Genetic Testing Results for Research and Direct-to-Consumer Applications

**DOI:** 10.3390/genes9100481

**Published:** 2018-10-04

**Authors:** Max Robinson, Gustavo Glusman

**Affiliations:** Institute for Systems Biology, 401 Terry Ave N, Seattle, WA 98109, USA; Max.Robinson@SystemsBiology.org

**Keywords:** computational genomics, genome comparison, algorithms, genetic testing, privacy, direct-to-consumer, study design, population genetics

## Abstract

Genetic testing has expanded out of the research laboratory into medical practice and the direct-to-consumer market. Rapid analysis of the resulting genotype data now has a significant impact. We present a method for summarizing personal genotypes as ‘genotype fingerprints’ that meets these needs. Genotype fingerprints can be derived from any single nucleotide polymorphism-based assay, and remain comparable as chip designs evolve to higher marker densities. We demonstrate that these fingerprints support distinguishing types of relationships among closely related individuals and closely related individuals from individuals from the same background population, as well as high-throughput identification of identical genotypes, individuals in known background populations, and de novo separation of subpopulations within a large cohort through extremely rapid comparisons. Although fingerprints do not preserve anonymity, they provide a useful degree of privacy by summarizing a genotype while preventing reconstruction of individual marker states. Genotype fingerprints are therefore well-suited as a format for public aggregation of genetic information to support ancestry and relatedness determination without revealing personal health risk status.

## 1. Introduction

A large number of genotypes have been produced by DNA hybridization, employing a variety of array designs [1]. The low cost of hybridization assays relative to sequencing, including whole-genome sequencing (WGS), exome sequencing, and other forms of targeted sequencing, has led to the commoditization of array-based genotyping and enabled commercial companies (including 23andMe, AncestryDNA, Family Tree DNA, and others [2]) to offer this service directly to consumers (DTC), which typically yield results with high concordance [3] and low no-call rates [4]. Genotyping the same individual using different array designs can yield slightly different results as each technology has its own biases. Even when using the same technology, genotype reference version, and variant encoding format, genotyping the same individual repeatedly can produce slightly different results due to the stochastic nature of genome processing and analysis, batch effects, or differences in the computational pipelines. Some companies regularly reanalyze the raw data for all customers, refining the results over time. As a result, customers who download their genotype data repeatedly over the years may have slightly differing results even from the same sample. In addition to relatedness applications, array-based genotyping is used as a quality control step prior to WGS when comprehensive variant information is desired.

Many methods exist for comparing genome-wide genotypes in order to infer relatedness with varying degrees of accuracy [5]. Most methods are computationally demanding and require full access to the genotype data of the individuals to be compared, potentially precluding their application to the study of samples with restricted access, or direct use of these methods by non-specialists interested in exploring their ancestry and genealogy.

A genotype determined by DNA hybridization enumerates all observed alleles for a predefined set of variants of interest—typically common single-nucleotide polymorphisms (SNPs). In this format, each SNP is identified by its identifier (reference SNP identifier, or rsid) in the dbSNP database [6]. For each rsid, the observed genotype of the individual is stated, including those for which the individual is homozygous for the reference allele. The chromosome and coordinate of the SNP, relative to a version of the reference that is (hopefully) stated in the ‘header’ of the genotype file, is implied by the rsid and not recorded in the genotype file.

We recently published a method for converting personal whole genome sequence data into ‘genome fingerprints’ that facilitate and accelerate their comparison [7]. Our method encodes the characteristics of pairs of consecutive single nucleotide variants (SNVs) relative to a reference, as represented in variant call format (VCF) files or structurally equivalent formats. In contrast to the format of genotyping results, WGS results in VCF format typically encode only differences from the reference. Genomic locations in which the individual is homozygous for the reference allele are typically not stated, achieving a more compact representation.

We present here an analogous method for summarizing personal genotypes, yielding genotype fingerprints that can be readily compared to estimate relatedness. The genotype fingerprints can be computed starting from any of several chip array designs, with genome coordinates expressed relative to any reference version. The resulting fingerprints are directly comparable without further conversion. Computation on the genotype fingerprints is fast and requires little memory, enabling comparison of large sets of genomes. No individual variants or other detailed features of the personal genome can be reconstructed from the fingerprint, allowing private information to be more closely guarded and protected, and enabling decoupling genome comparison from genome interpretation. Fingerprints of different sizes allow balancing the speed and accuracy of the comparisons. Due to the high value of estimating relatedness, the potential applications of genotype fingerprinting range from basic science (study design, population studies) to personalized medicine, forensics, and data privacy. 

## 2. Materials and Methods 

### 2.1. Methodology Overview

**Figure 1:**
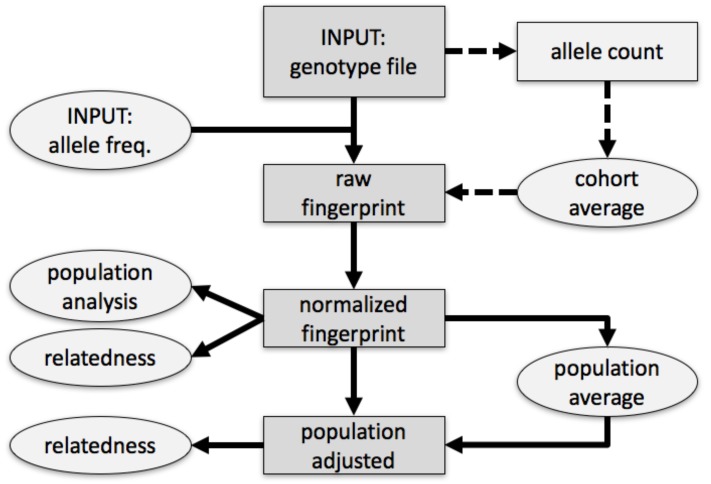
Overview of method. Single nucleotide polymorphisms (SNPs) in the input genotype file were encoded into a table (raw) by observed alleles and rsid numerical value, considering allele frequencies. This could optionally be approximated by subtracting allele counts estimated from a simple model of an observed cohort (dashed arrows). The raw fingerprint was then normalized and could be adjusted to represent deviation from the center of the closest population. Rectangles and ellipses pertain to individual genotypes or to multiple genotypes, respectively; darker gray denotes the flow of information for one genotype from the input file to the normalized and adjusted fingerprints.

We fingerprinted genotypes in four stages. First, we summarized a genotype as a tally of biallelic SNPs, stratified by observed alleles, by variant identifiers, and accounting for allele frequencies (raw fingerprint, Figure 1). We then normalized the raw fingerprint to account for systematic methodological patterns. The resulting normalized fingerprint preserves differences between individuals from different groups (populations), and are appropriate for clustering individuals by population. To determine whether individuals were assigned to populations a priori or via clustering, we averaged the normalized fingerprints of the individuals in a population to produce a ‘population’ fingerprint, which characterizes the population rather than an individual. To improve detection of relationships within a population, we derived a ‘population-adjusted’ fingerprint from an individual’s normalized fingerprint by subtracting the associated population fingerprint. Documentation, code, and sample datasets are available at the project’s website. [8].

### 2.2. Raw Fingerprints

A raw fingerprint is a 4-row by *L*-column table of SNP allele counts, where *L* is the main parameter of the method and determines the information content of the fingerprint. By default, *L* = 1000. The four rows correspond to the permitted alleles of binary SNPs (A, C, G and T); variants with other possible alleles (including insertions, deletions, and multi-nucleotide variants) were ignored, partly because binary SNPs are so abundant but also because other variant classes are subject to more variation in how each observable allele is reported. We tallied all observed alleles for each SNP (reference and alternate). Each SNP was tallied in a column determined from its rsid, which is its reference number in the dbSNP database [6]. The full process is as follows:

(1) Filter out all variants that are not autosomal, biallelic SNPs with reference and alternate alleles limited to A, C, G, and T. Optionally, include only SNPs in a preselected set, e.g., 23andMe (Mountain View, California, USA) V2 and V3. The remaining steps are applied to each retained SNP.
(2) Determine a fingerprint column as the rsid modulo *L* (when *L* = 1000, SNP rs1801133 will be recorded in column 133).
(3) For each SNP, count observations of each nucleotide: n_A_ is the count of A alleles (0, 1, or 2), n_C_ is the count of C alleles, etc., with n_N_ = n_A_ + n_C_ + n_G_ + n_T_ = 2.
(4) Determine the expected count E[n_X_] = n_N_ f_X_ for each nucleotide X from a set of known allele frequencies f_A_ + f_C_ + f_G_ + f_T_ = 1. Depending on the context, these frequencies may be specific to each SNP (e.g., for human data, extracted from dbSNP), or without reference to external data, and may be computed per column from all SNPs contributing to the column among an observed cohort of genotypes (detailed below).
(5) In the column for the SNP’s rsid, tally differences n_X_ − E[n_X_] from expectation for each row (add n_A_ − E[n_A_] to the row for A, n_C_ − E[n_C_] to the row for C, etc.).

Retrieving the allele frequencies for each observed rsid requires prior knowledge and can incur significant computational costs. A more efficient variant involves computing expected frequencies for each row and column directly from a cohort of genotypes of a common type (determined with the same assay, array design, etc.) as follows:

(1) Tally allele counts separately for each individual as above, except in step 5, increase the value in the [4 × *L*] matrix by 1 for each observed allele. In case of homozygosity, increasing twice results in an increase by 2. These tallies result in summed N_A_, N_C_, N_G_, and N_T_ values in each column, with column total N_N_ = N_A_ + N_C_ + N_G_ + N_T_ = 2k, twice the number of SNPs assigned to the column. The steps below do not require reprocessing the full genotypes, only these 4 × *L* tallies per individual.
(2) Compute cohort average frequencies by summing the tallied allele counts in each column across all individuals and dividing by the column total. This produces four expected allele frequencies E[f_X_] for each column, and corresponding expected tallies E[N_X_] = N_N_ E[f_X_].
(3) Finish computing each entry in each individual’s raw fingerprint by subtracting the expected tallies, t_X_ = N_X_ − E[N_X_].

### 2.3. Fingerprint Normalization

In this step, we accounted for unequal assortment of genotype information within the fingerprint. This imbalance was due to a methodological aspect of the fingerprinting process (grouping of variants by rsid), not a source of information of the fingerprinted individuals.

(1) We subtracted the mean and divided by the standard deviation of each column, which mitigated differences between columns in the types of nucleotide substitutions (transitions and transversions), which were derived from the set of rsids assigned to each column.
(2) We then subtracted the mean and divided by the standard deviation of each row. This further mitigated methodological differences between values within the fingerprint, which primarily reflect the genotyping methodology rather than variation between individuals.

### 2.4. Population Fingerprints

We computed a population fingerprint as the average of the normalized fingerprints from the individuals in the population. Note that genotype fingerprints are only directly comparable when computed using the same format parameter *L*; different values of *L* cause rsids to be grouped into columns differently. However, different versions of a genotype array design contain substantial overlaps in the set of SNPs included in the array, and rsids are grouped in the same manner for a given value of *L* regardless of array design. Thus, genotypes from the same population on slightly different variants of the same array design may be mixed in computing the population fingerprint.

### 2.5. Adjusting Fingerprints for Population

We then computed a population-adjusted fingerprint for an individual by subtracting a population fingerprint from the normalized fingerprint of that individual. These individual fingerprints and the population fingerprint must have been computed using the same parameter *L*.

### 2.6. Fingerprint Comparison

To compare two fingerprints, concatenate the rows of each fingerprint matrix into a vector and compute the Spearman correlation between the two vectors. This same procedure was appropriate for comparing two normalized fingerprints or two population-adjusted fingerprints, whether adjusted to the same or different populations.

### 2.7. Family Analyses

We obtained 23andMe SNP chip genotype data for a family of five [9], including mother, father, son, daughter, and aunt. The son was 23andMe V2 data and the rest of the family were 23andMe V3 data. We computed normalized genotype fingerprints (*L* = 5000) for the five individuals and performed all pairwise comparisons. We also extracted from these samples the lists of rsids observed in V2 and V3 for use in further analyses.

We also studied WGS data from 35 individuals in a large family (Figure S1). All genomes were sequenced by Complete Genomics, Inc. (Mountain View, California, USA) from blood (*n* = 25) or saliva (*n* = 10) samples, and processed using pipeline version 2.5.0.20. The genome data and a description of the family pedigree were donated by the private family. For each individual, we extracted observed genotypes twice, once for each rsid in the 23andMe V2 SNP list and once using the V3 SNP list. We then computed genotype fingerprints (*L* = 5000) from these extracted genotypes. We categorized all pairwise relationships within the family as sibling, parent/child, half-sibling, aunt/uncle, grandparent, cousin, second cousin, second cousin once removed, and unrelated. We excluded more complex relations, e.g., child and grandchild of half-siblings. We computed all pairwise correlations using the same chip design (e.g., both individuals in the pair using the V2 SNP list) and using discordant chip designs (e.g., one individual with V2 and the other with V3). Finally, we computed the average and standard deviation of the computed correlations, stratified by the relationship categories listed above.

### 2.8. Population Structure Analysis

Principal components analysis (PCA) is a standard method for characterizing population structure prior to genome-wide association studies (GWAS). We therefore compared well-characterized population structures within data from the 1000 Genomes Project (release 20130502, ALL.chrNN.phase3_shapeit2_mvncall_integrated_v5.20130502.genotypes.vcf.gz). As a genomic method, we identified SNPs with a minor allele frequency of 5% or more, removed SNPs in complete linkage disequilibrium with a SNP to the left (i.e., a smaller chromosomal position), retained 5% at random (298,454 SNPs), and counted occurrences of the minor allele (0, 1, or 2) in each genome to form a 2504 × 298,454 genotype matrix M.

As a fingerprint-based method, we extracted observed genotypes for each of the 2504 genomes twice, once for each rsid in the 23andMe V2 SNP list and once using the V3 SNP list. We then computed genotype fingerprints from these extracted genotypes using *L* = 500, 1000, and 5000, resulting in fingerprint data matrices of 2504 × 2000, 2504 × 4000, and 2504 × 20,000 entries, respectively. We performed standard PCA separately on the six resulting matrices (V2 or V3 and *L* = 500, 1000, or 5000) using the R function call prcomp(M,center=TRUE,scale.=TRUE).

### 2.9. Evaluation of “Nearest Population Fingerprint” for Population Assignment

We computed a population fingerprint for each of the 26 populations selected for study in the 1000 Genomes Project. We then re-classified each individual via fingerprint comparison against the 26 population fingerprints, as described above for comparing individual fingerprints. Each individual was classified as belonging to the population with the closest population fingerprint. To avoid overfitting, we excluded each individual from the computation of their own population fingerprint in leave-one-out fashion. 

## 3. Results

### 3.1. Method for Encoding Genotyping Data

We developed a locality sensitive hashing [10] algorithm for computing fingerprints from genotype data, including data produced by a DTC genetics company (23andMe, Mountain View, California, USA). These genotype fingerprints meet the characteristics of genotype data: they can be rapidly computed starting from any of several chip array designs, with genome coordinates expressed relative to any reference version, and the resulting fingerprints are directly comparable as long as the same fingerprint length *L* is used. We described fingerprints generated using SNP lists derived from two array designs used by 23andMe: V2, based on Illumina (Foster City, California, USA) HumanHap550 Genotyping BeadChip (~550,000 SNPs) and V3, based on Illumina OmniExpress Genotyping BeadChip (~960,000 SNPs). The fingerprints are a reduced representation of the genotype data computed once per individual, and can be efficiently databased and compared to determine whether two genotypes represent the same individual, closely related individuals, or unrelated individuals. As with our previously reported genome fingerprints [7], individual alleles cannot be reconstructed from the genotype fingerprint beyond what is predictable from detectable population and family relationships, enabling sharing of fingerprints for comparison when privacy concerns prevent sharing the full genotype file itself.

The main parameter of our algorithm, *L*, determines the size and SNP groupings of the fingerprint. Smaller fingerprints (e.g., *L* = 100) average variants over fewer bins and are useful for extremely fast, low-resolution comparisons, to determine identity, whereas larger fingerprints (*L* = 1000 or 5000) are higher resolution and better support detailed analyses including population reconstruction.

### 3.2. Computation on Genotype Fingerprints is Fast

Generation of raw genotype fingerprints is simple, and its speed is limited by the time required to read the file rather than the fingerprint size *L*. On our workstations, it required 10–15 s per 500,000–960,000-SNP genotype. For higher speeds, each raw fingerprint is independent and the process is readily parallelized. In our population studies, population fingerprints for all 26 populations of the 1000 Genomes cohort required less than 60 s, fingerprint normalization averaged 0.13 s per genotype, and serializing the 2504 fingerprints for efficient comparison required only 37 s. The 3,133,756 “all-against-all” comparisons for this data set took 15 CPU seconds for *L* = 1000 (4.8 microseconds per comparison) and 79 CPU seconds for *L* = 5000 (25.2 microseconds per comparison). Although parallelization was not required for this cohort, it is straightforward and may be helpful for all-against-all comparisons of cohorts of tens to hundreds of thousands of genotypes.

### 3.3. Rapid Relationship Detection

**Figure 2:**
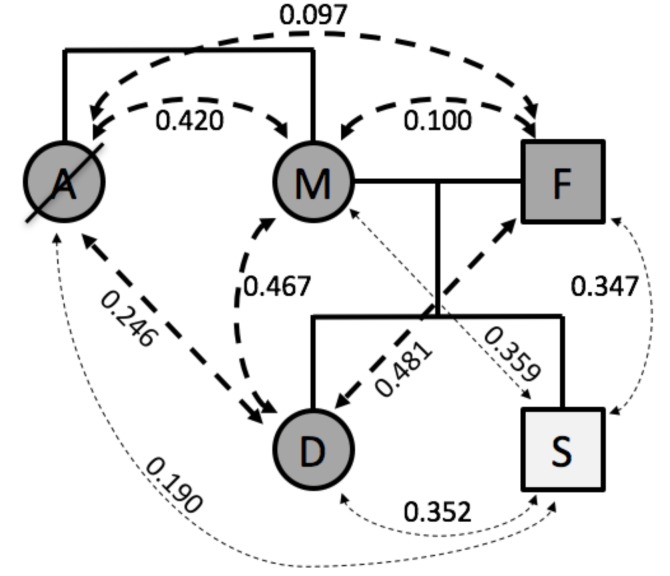
Comparison within a family of five. A: Aunt (deceased); M: Mother; F: Father; D: Daughter; S: Son. Dashed lines represent family relationships; thin lines denote comparison between individuals assayed on different versions of the genotyping platform.

Here, we illustrate the use of genotype fingerprints for characterizing family relationships within a family of five [4] who had previously made their 23andMe genotype results publicly available. Comparisons of these fingerprints resulted in similarity scores (Spearman’s rho values) that were consistent with the known family relationship types (Figure 2). Rho values for full sibling pairs (aunt and mother 0.420, daughter and son 0.352) and parent-offspring pairs (0.481, 0.467, 0.359, and 0.347) were higher than for avuncular relationships (aunt and daughter 0.246, aunt and son 0.190), which in turn were higher than unrelated pairs (aunt and father 0.097, mother and father 0.100).

The correlations between the son and the other family members was reduced, as expected for comparisons across SNP lists (V2 for son, V3 for all others) with both substantial overlap and differences. To further quantify this effect, we compared genotype fingerprints based on both chip designs (V2 and V3) in the context of a larger family pedigree (Figure S1). We observed a ratio of 0.75 when comparing pairwise correlations of fingerprints based on discordant designs (i.e., V2–V3) vs. correlations of fingerprints based on the same design, i.e., V2–V2 and V3–V3 (Figure S1C). It is thus possible to adjust by scaling the correlations observed from discordant designs (Figure S1D), yielding comparable correlations for each relationship level independent of chip design.

### 3.4. Rapid Analysis of Population Structure

**Figure 3:**
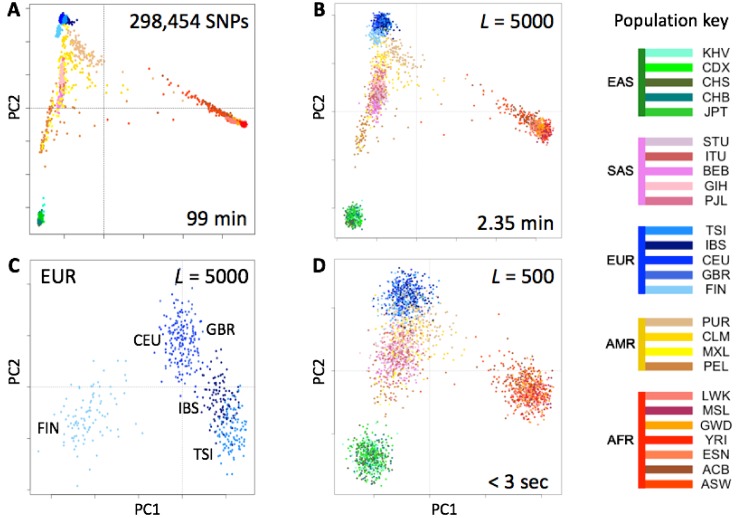
Estimates of population structure in the 1000 Genomes Project data set at different resolutions. Individuals are color coded according to their population as per the key to the right. EAS, SAS, EUR, AMR and AFR: East Asian, South Asian, European, Admixed American, and African, respectively. (**A**) Principal components analysis (PCA) of the 2504 individuals using ~300,000 SNPs. (**B**) PCA on genotype fingerprints (N = 2504) with *L* = 5000. (**C**) PCA on genotype fingerprints of European populations (N = 503) with *L* = 5000. (**D**) PCA on genotype fingerprints (N = 2504) with *L* = 500.

We tested the utility of genotype fingerprints for population studies. We extracted genotypes for all 2504 individuals of the 1000 Genomes Project cohort from their VCF-format genomes using the 23andMe V3 SNP list, fingerprinted each extracted genotype, and used PCA to reconstruct the known population structure (Figure 3). This entire process required a fraction of the time and memory to perform the same task using a more standard approach (see Methods). As expected, the quality of the reconstruction depended on fingerprint resolution (*L*): fingerprints with *L* = 5000 yielded excellent population structure reconstruction, comparable to the results of population reconstruction using high-resolution genome fingerprints (compare Figures 3 and 5 from Glusman et al. [7]). Genotype fingerprints with smaller values of *L* yielded progressively lower-resolution results at proportionally higher speeds (seconds rather than more than an hour, Figure 3). Analysis of only the closely related European populations (Figure 3C) showed the same separations apparent in analyses of a limited number of SNPs (not shown): full separation of the Finns (FIN), the Northwestern Europeans: Utah Residents with Northern and Western European Ancestry (CEU), British in England and Scotland (GBR), and the Mediterraneans: Iberians in Spain (IBS), Toscani in Italy (TSI), with less separation of IBS from TSI and essentially none between CEU and GBR. Thus, genotype fingerprints retain differences at both large and small scales, allow a suitable balance between resolution and speed, and support scaling of population structure studies to whole population-scale genotype data, without requiring analysis of linkage disequilibrium and other complications prior to analysis.

### 3.5. Rapid Population Assignment

**Figure 4:**
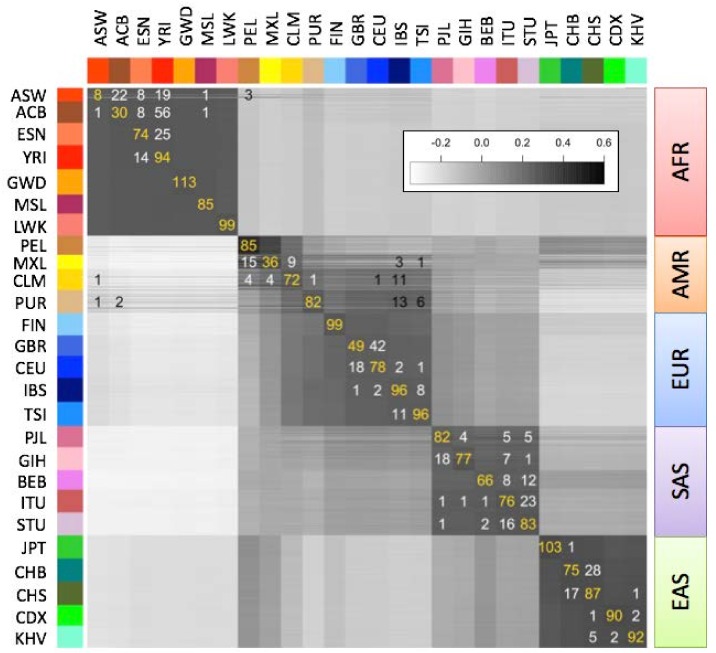
Correlations between the genotype fingerprints of the 2504 individuals (rows) and the average fingerprints of the 26 populations (columns) in the 1000 Genomes Project. Population codes and colors as in Figure 3. Numbers in gold, white, and black denote population assignments: to the same annotated population, to the same continent but different population, or to a different continent, respectively. Inset: correlation color scale.

We computed “population fingerprints” in the 1000 Genomes data set by averaging genotype fingerprints (V3 set, *L* = 5000) of the individuals in each population. To determine each individual’s population of origin, we then computed the correlation between the fingerprint of a query genome and the fingerprint of each population (Figure 4), and classified each individual as belonging to the population with the strongest fingerprint correlation. We evaluated this classification method using leave one out cross-validation. The annotated population had the highest correlation for 2027 of the 2504 samples (81%), or among the top two- (92.9%) or top three-most (96.1%) correlated. The only misclassifications to a population from another continent involved the Admixed American populations (AMR); excluding these populations increased correct classifications to 85.7% (best match), 97.8% (top two) and 99.2% (top three). In general, misclassifications both between and within continental groups occurred between historically or geographically associated population pairs: Americans of African Ancestry in Southwest USA (ASW) and African Caribbeans in Barbados (ACB) with Nigerian populations Esan (ESN) and Yoruban (YRI); Latin American populations Mexican Ancestry from Los Angeles (MXL), Colombians from Meellín (CLM), and Puerto Ricans (PUR) with Mediterranean populations IBS, TSI; Northern and Southern Han Chinese populations CHB, CHS; South Indian populations Indian Telugu from the UK (ITU), Sri Lankan Tamil from the UK (STU), suggesting that admixture was the principal source of misclassification. Even for very closely related population pairs (e.g., CEU and GBR), individuals were more often correctly classified than misclassified.

### 3.6. Robustness to SNP List

**Figure 5:**
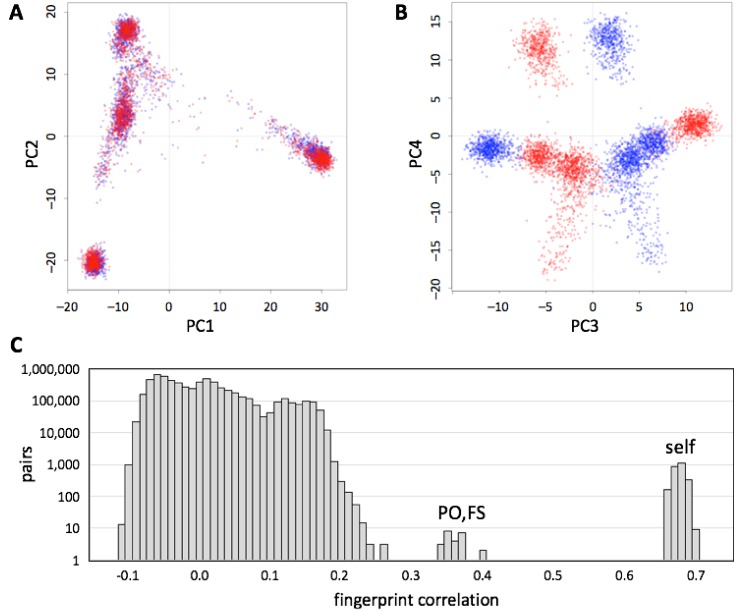
Comparison of genotype fingerprints relative to different SNP lists. We deduced normalized genotype fingerprints (*L* = 5000) for the 1000 Genomes Project cohort using the 23andMe V2 (red) and V3 (blue) SNP lists. (**A**) First two principal components, showing population structure. (**B**) Third and fourth principal components, showing separation between the two SNP lists. (**C**) Distribution of cross-correlations between the two sets of genotype fingerprints (all possible pairs of V2 vs. V3). Comparisons between the two genotype fingerprints for the same individual (self) and comparisons between parent/offspring and full-sibling pairs (PO, FS) formed distinct, high-correlation subsets.

We evaluated whether genotype fingerprints can be compared across chip array designs. We fingerprinted genotypes for each individual in the 1000 Genomes Project data set, extracted using the 23andMe V2 and V3 SNP lists (548,911 and 902,448 SNPs, respectively), yielding a mixed set of 5008 fingerprints (V2 and V3 fingerprints for each of 2504 individuals). We studied this joint set using PCA (Figure 5A) and observed that the first two principal components reconstruct the known population structure (Figure 3). PC3 separated between fingerprints computed on V2 and V3 versions (Figure 5B). The correlations between the two versions of each individual (self, Figure 5C) were always higher than those between related individuals (parent/offspring, full siblings), which in turn were higher than those between unrelated individuals. 

We further evaluated the robustness of genotype fingerprints for population assignment across array designs, for example, when the individual being studied was genotyped on a different array than the populations being used as reference. As above, we classified each of the individuals in the 1000 Genomes data set using genotype fingerprints (*L* = 5000) based on the V2 or the V3 sets, and relative to population fingerprints derived from the V2 or the V3 sets. All four combinations yielded similar classifications (Figure S2). As expected, the classifications using discordant SNP lists were slightly less accurate.

Although fingerprints derived using different versions of the same chip design were distinguishable, comparisons between them would still be useful for detecting identical individuals, family analysis (Section 3.3.), and population analysis.

### 3.7. Fast Detection of Close Relationships

**Figure 6:**
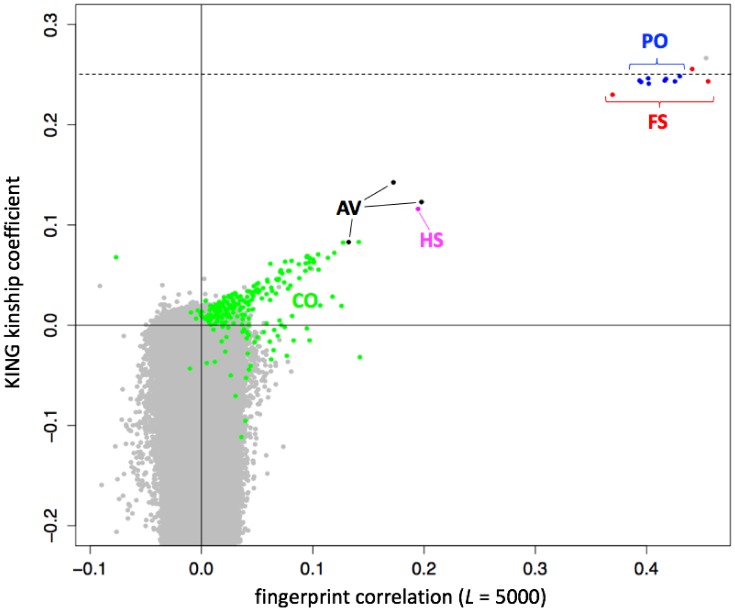
Identification of close relationships in the 1000 Genomes Project. Comparison between the correlations of population-adjusted genotype fingerprints (V3 set, *L* = 5000) and the kinship coefficient computed using KING, highlighting close relationships identified using RELPAIR. FS: full siblings (red). PO: parent/offspring (blue). HS: half siblings (magenta). AV: avuncular (black). CO: cousins (green). All other pairs in gray. One FS pair (HG03873 and HG03998, with maximal kinship, in gray) was not identified by RELPAIR.

Based on our work with genome fingerprints, we reasoned that using population fingerprints to cancel out correlations due to information shared among a population would allow close relationships to be distinguished from shared population backgrounds. We therefore adjusted the *L* = 5000 V3-type fingerprints (see Section 3.6.) for their annotated population of origin and performed all pairwise comparisons of these adjusted fingerprints. We compared the fingerprint correlations with kinship coefficients computed using KING [11] and with previously reported relationships [12] computed using RELPAIR [13] (Figure 6). As expected, the correlation between individuals from the same annotated population (Figure 4), but not related within a few generations, was removed by adjustment to the population average (Figure 6), and population-adjusted fingerprints for unrelated individuals were essentially uncorrelated. Comparison of population-adjusted genotype fingerprints supports the detection of individuals in the 1000 Genomes cohort previously reported as closely related [12]. The highly-correlated pairs correspond to relationships of degrees varying from full siblings to cousins. For these pairs, fingerprint correlations showed a linear relationship with kinship coefficients computed by KING (Figure 6, colored points). Parent-offspring and full sibling relationships, which have the same expected KING kinship coefficient (0.25) but different variance from that expected value, produced equivalent high fingerprint correlations of around 0.4.

## 4. Discussion

We presented a method for computing fingerprints of genome-wide SNP array genotypes as reported by DTC genetics companies, using 23andMe data as an example. Like our previously reported fingerprints from whole-genome resequencing data, genotype fingerprints retain sufficient information to enable ultrafast comparison of genotypes, without retaining the sensitive individual SNP data necessary to predict phenotypes. Genotype fingerprints are therefore suitable for databasing and sharing ancestry and close relationship determination without exposing more sensitive health-related information.

We demonstrated the utility of genotype fingerprints for rapid versions of common tasks: identifying genotypes from the same individual, from closely related individuals, or from a known population, and de novo clustering of individuals into subpopulations. Comparing fingerprints derived from two different SNP lists (23andMe V2 and V3), our genotype fingerprints were robust to differences in the number of SNPs assayed for detecting identity, detecting close relationships, and delineating populations.

Conceptually, genotype fingerprints are an adaptation of our genome fingerprinting method [7] to more widely available, more standardized, but lower-resolution genotype data. Whereas genome fingerprints facilitate comparison of data across different reference sequence versions by encoding consecutive SNV pairs, genotype fingerprints achieve a similar interoperability by encoding individual SNPs using annotated rsids, alleles, and allele frequencies. SNPs are simply SNVs with high population frequency, but this frequency difference has practical consequences. Although whole genome sequencing is expected to reveal an increasing number of rare SNVs, the vast majority of SNPs have already been identified, evaluated for linkage, assigned stable identifiers (rsids), and incorporated into high-throughput assays. In contrast, many SNVs either lack identifiers or have been assigned preliminary identifiers still subject to change (e.g., by merging with a different identifier representing the same variant). Stable identifiers facilitate matching variants across genome reference versions and assays, enabling the desired robustness to a changing reference genome using the simpler encoding method presented here.

Insertions and deletions have also been assigned standard representations in genotype files (symbols I and D, respectively), but are much less abundant in the genome than SNPs, are not as widely assayed, and require normalization prior to extraction as genotypes from WGS or exome data [14]. For simplicity and consistency, we therefore chose to exclude them from analysis, as we did for computing genome fingerprints from VCF files. We also chose to exclude SNPs on the sex chromosomes, which vary in count between males and females and may lead to distorted similarity values.

Sharing genetic information raises several privacy concerns. Much attention has been paid to the risk of re-identification of de-identified samples [15], even when querying genetic data sets via bandwidth-limiting interfaces like the GA4GH beacons. These concerns have given rise to privacy preservation strategies such as obscuring rare variants and budgeting queries [16]. While enabling an important and powerful query, namely, “has this allele been seen before?” [17], these strategies for preventing re-identification preclude multiple other potential applications, thus limiting the utility of genome data sharing. There are, however, genetic data sharing scenarios in which anonymity is not, but phenotype prediction is, an issue. For example, an individual may wish to compare their genotype (obtained via a DTC genetic testing company) to the genotypes of other individuals for ancestry and relationship determination, but without revealing whether their genome harbors alleles associated with a specific phenotype (e.g., Alzheimer’s disease) both currently known alleles and ones whose significance may be discovered in the future. Like genome fingerprints, genotype fingerprints decouple genotype comparison from genotype interpretation, supporting the identification of closely related individuals without exposing individual variant states.

At present, the number of private individuals who have used DTC genetics services to ascertain their own genotype vastly exceeds the number of individuals with full genome data. We expect genotype fingerprints to have immediate applicability for facilitating genotype comparisons, empowering citizen science without concomitantly revealing sensitive private genetic information.

## Supplementary Materials

The following are available online at the project website [8], and at this link: http://www.mdpi.com/2073-4425/9/10/481/s1, Figure S1: Genotype fingerprints analysis of larger family pedigree, Figure S2: Correlations between the genotype fingerprints of the 2504 individuals (rows) and the average fingerprints of the 26 populations (columns) in the 1000 Genomes Project.

## References

[1] Canada R. Exploring Microarray Chips. http://haplogroup.org/exploring-microarray-chips/.

[2] List of DNA testing companies—ISOGG Wiki. https://isogg.org/wiki/List_of_DNA_testing_companies.

[3] Imai K., Kricka L.J., Fortina P. (2011). Concordance study of 3 direct-to-consumer genetic-testing services. Clin. Chem..

[4] Glusman G., Cariaso M., Jimenez R., Swan D., Greshake B., Bhak J., Logan D.W., Corpas M. (2012). Low budget analysis of Direct-To-Consumer genomic testing familial data. F1000 Research.

[5] Ramstetter M.D., Dyer T.D., Lehman D.M., Curran J.E., Duggirala R., Blangero J., Mezey J.G., Williams A.L. (2017). Benchmarking relatedness inference methods with genome-wide data from thousands of relatives. Genetics.

[6] Sherry S.T., Ward M.H., Kholodov M., Baker J., Phan L., Smigielski E.M., Sirotkin K. (2001). dbSNP: The NCBI database of genetic variation. Nucleic Acids Res..

[7] Glusman G., Mauldin D.E., Hood L.E., Robinson M. (2017). Ultrafast comparison of personal genomes via precomputed genome fingerprints. Front. Genet..

[8] Genotype fingerprints’ homepage. http://db.systemsbiology.net/gestalt/genotype_fingerprints.

[9] Glusman G., Cariaso M., Jimenez R., Swan D., Greshake B., Bhak J., Logan D.W., Corpas M. 23andMe SNP chip genotype data 2012. https://figshare.com/articles/23andMe_SNP_chip_genotype_data/92682.

[10] Indyk P., Motwani R. (1998). Approximate nearest neighbors. Proceedings of the thirtieth annual ACM symposium on Theory of computing—STOC ’98;.

[11] Manichaikul A., Mychaleckyj J.C., Rich S.S., Daly K., Sale M., Chen W.-M. (2010). Robust relationship inference in genome-wide association studies. Bioinformatics.

[12] Gazal S., Sahbatou M., Babron M.-C., Génin E., Leutenegger A.-L. (2015). High level of inbreeding in final phase of 1000 Genomes Project. Sci. Rep..

[13] Epstein M.P., Duren W.L., Boehnke M. (2000). Improved inference of relationship for pairs of individuals. Am. J. Hum. Genet..

[14] Tan A., Abecasis G.R., Kang H.M. (2015). Unified representation of genetic variants. Bioinformatics.

[15] Erlich Y., Williams J.B., Glazer D., Yocum K., Farahany N., Olson M., Narayanan A., Stein L.D., Witkowski J.A., Kain R.C. (2014). Redefining genomic privacy: Trust and empowerment. PLoS Biol..

[16] Raisaro J.L., Tramèr F., Ji Z., Bu D., Zhao Y., Carey K., Lloyd D., Sofia H., Baker D., Flicek P., Shringarpure S., Bustamante C., Wang S., Jiang X., Ohno-Machado L., Tang H., Wang X., Hubaux J.-P. (2017). Addressing beacon re-identification attacks: Quantification and mitigation of privacy risks. J. Am. Med. Inform. Assoc..

[17] Glusman G., Caballero J., Mauldin D.E., Hood L., Roach J.C. (2011). Kaviar: An accessible system for testing SNV novelty. Bioinformatics.

