# Supplementary material for: Genotype Fingerprints Enable Fast and Private Comparison of Genetic Testing Results for Research and Direct-to-Consumer Applications
Source: Genes (Basel). 2018 Oct 4;9(10):481. doi: 10.3390/genes9100481 (PMC6209914; doi:10.3390/genes9100481)

genes

MDPI

Supplementary Material

Genotype fingerprints enable fast and private comparison of genetic testing results for research and direct-to-consumer applications

Max Robinson and Gustavo Glusman*

*** Correspondence:** Gustavo Glusman: Gustavo@SystemsBiology.org

Supplementary Figure 1. Genotype fingerprints analysis of larger family pedigree. (A) Family structure. Individuals sequenced are labeled in green. (B) Number of pairs of individuals observed for different relationship distances. (C) Spearman rho vs. relationship distance when comparing fingerprints from the same chip design (circles) or different designs (crosses), and the ratio between them (triangles). (D) Spearman rho vs. relationship distance after scaling.

A

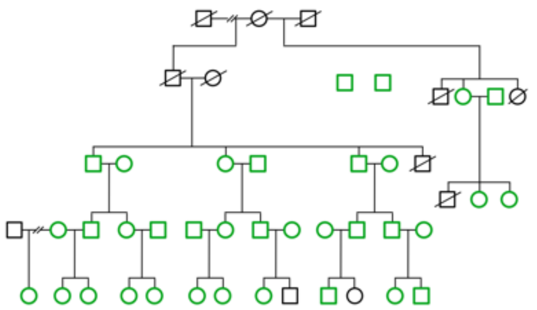

B

Relationship	N
self	35
sibling	12
parent/child	39
half-sibling	2
avuncular	25
grandparent	22
great uncle/aunt	23
cousin	24
second cousin once removed	46
second cousin	40
unrelated	297

C

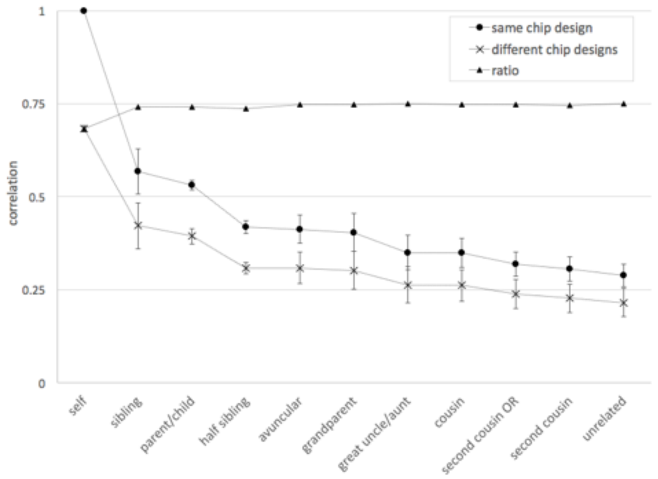

D

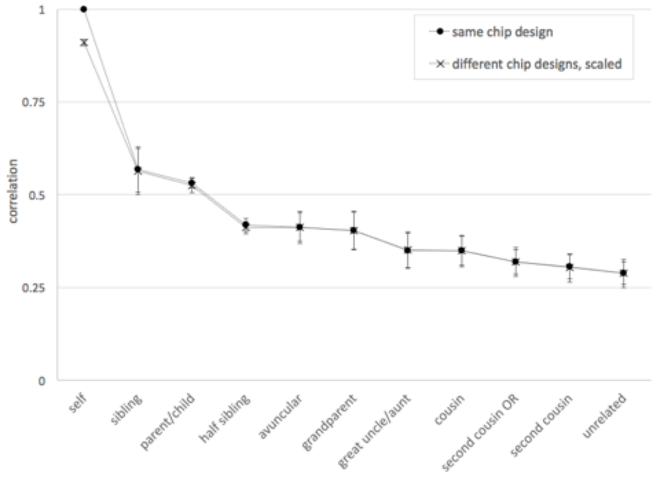

Supplementary Figure 2. Correlations between the genotype fingerprints of the 2504 individuals (rows) and the average fingerprints of the 26 populations (columns) in the 1000 Genomes Project, contrasting SNP lists V2 and V3 for the individuals and the populations. Population codes and colors as in Figures 3 and 4. Numbers in gold, white and black denote population assignments: to the same annotated population, to the same continent but different population, or to a different continent, respectively. Larger inset numbers denote totals for each of the three classification success levels.

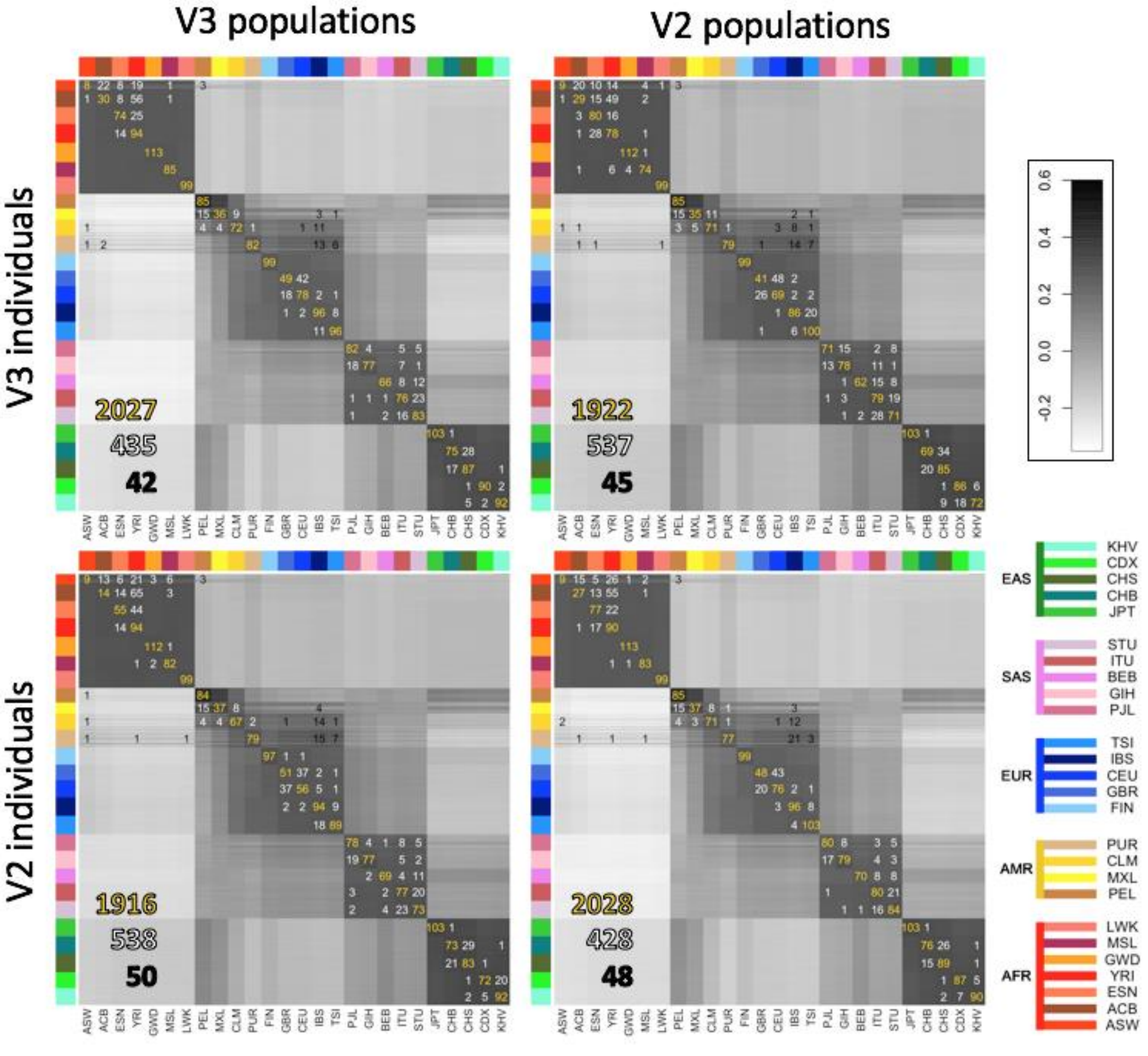

Supplement: Supplementary file 1 [file genes-09-00481-s001.pdf]
